# Claudins: Beyond Tight Junctions in Human IBD and Murine Models

**DOI:** 10.3389/fphar.2021.682614

**Published:** 2021-11-17

**Authors:** Snježana Čužić, Maja Antolić, Anja Ognjenović, Darija Stupin-Polančec, Adriana Petrinić Grba, Boška Hrvačić, Miroslava Dominis Kramarić, Sanja Musladin, Lidija Požgaj, Ivo Zlatar, Denis Polančec, Gorana Aralica, Marko Banić, Marija Urek, Brankica Mijandrušić Sinčić, Aleksandar Čubranić, Ines Glojnarić, Martina Bosnar, Vesna Eraković Haber

**Affiliations:** ^1^ Fidelta, Zagreb, Croatia; ^2^ School of Medicine, University Zagreb, Zagreb, Croatia; ^3^ Department of Pathology Clinical Hospital Dubrava, Zagreb, Croatia; ^4^ Department of Internal Medicine Clinical Hospital Dubrava, Zagreb, Croatia; ^5^ Faculty of Medicine, University of Rijeka, Rijeka, Croatia; ^6^ Department of Internal Medicine, Clinical Hospital Center Rijeka, Rijeka, Croatia

**Keywords:** claudins, inflammatory bowel disease, dextran sodium sulfate colitis model, adoptive transfer colitis model, ulcerative colitis, Crohn’s disease

## Abstract

Claudins are transmembrane proteins constituting one of three tight junction protein families. In patients with inflammatory bowel disease (IBD), disease activity–dependent changes in expression of certain claudins have been noted, thus making certain claudin family members potential therapy targets. A study was undertaken with the aim of exploring expression of claudins in human disease and two different animal models of IBD: dextrane sulfate sodium–induced colitis and adoptive transfer model of colitis. The expression of sealing claudin-1, claudin-3, claudin-4, and claudin-8, and pore-forming claudin-2 in humans and rodents has been evaluated by immunohistochemistry and quantitative polymerase chain reaction. Claudins were expressed by epithelial and cells of mesodermal origin and were found to be situated at the membrane, within the cytoplasm, or within the nuclei. Claudin expression by human mononuclear cells isolated from lamina propria has been confirmed by Western blot and flow cytometry. The claudin expression pattern in uninflamed and inflamed colon varied between species and murine strains. In IBD and both animal models, diverse alterations in claudin expression by epithelial and inflammatory cells were recorded. Tissue mRNA levels for each studied claudin reflected changes within cell lineage and, at the same time, mirrored the ratio between various cell types. Based on the results of the study, it can be concluded that 1) claudins are not expressed exclusively by epithelial cells, but by certain types of cells of mesodermal origin as well; 2) changes in the claudin mRNA level should be interpreted in the context of overall tissue alterations; and 3) both IBD animal models that were analyzed can be used for investigating claudins as a therapy target, respecting their similarities and differences highlighted in this study.

## Introduction

Tight junctions (TJs), together with adherens junctions and gap junctions, constitute the intestinal epithelial barrier that regulates paracellular permeability (Lee et al., 2018). Apart from building up TJs, TJ proteins are involved in regulation of other cell functions, such as maintenance of cell polarity, proliferation, and differentiation (Farkas et al., 2012).

Inflammatory bowel disease (IBD), which comprises Crohn disease (CD) and ulcerative colitis (UC), is characterized by damage of the intestinal epithelium and prolonged chronic inflammation. Altered TJ function and TJ-protein expression in IBD have attracted the attention of the scientific community due to increased permeability of the intestinal epithelial barrier being one of the hallmarks of IBD, presenting even before onset of overt inflammation in CD patients (Chelakkot et al., 2018; Keita et al., 2018).

As rising incidence of IBD (Ng et al., 2017) has kindled significant drug discovery research efforts aimed at controlling IBD clinical manifestations, animal models of IBD play an important role in the testing cascade of new therapeutics. The aim of this study was to evaluate the potential use of animal models of intestinal inflammation in studying expression of claudins and constitutive TJ proteins, relevant for IBD in humans. Colon expression of sealing claudin-1, claudin-3, claudin-4, and claudin-8 and pore-forming claudin-2 in IBD patients, IBD models, and their nondiseased controls was examined.

Animal models of two different pathophysiology were chosen for the study. The first model was dextrane sulfate sodium (DSS)–induced colitis caused by epithelial barrier disruption, leading to antigen leakage and finally inflammation (Pickert et al., 2009). The second model was adoptive transfer colitis induced by transfer of CD4^+^CD25^−^CD62L^+^ T lymphocytes into immunodeficient mice causing T_H_17-mediated mucosal inflammation and reactive epithelial proliferation (Mars et al., 2009; Brockman et al., 2017; Kempski et al., 2017).

## Materials and Methods

### Patients

Formalin-fixed paraffin-embedded (FFPE) colon samples were obtained from IBD patients who underwent small intestine/colon resection due to the severity of disease. There were eight patients with CD (four female and four male patients, 27–84 years of age [average age, 45 years]) and five patients with UC (four female and one male patients, 18–51 years of age [average age, 38 years]). Control FFPE colon samples were obtained from patients undergoing colon resection due to carcinoma (eight non-IBD control subjects: one female and seven male control subjects, 50–90 years of age [average age, 72 years]). Disease activity in IBD patients was evaluated using a published histologic score method (Naini and Cortina, 2012), excluding neuroendocrine hyperplasia (Supplementary Table S1A). Human tissue specimens used for lamina propria mononuclear cell (LPMC) isolation for Western blot and flow cytometry analysis included surgical colon resections and mucosal biopsies from 5 UC patients (four female and one male patients, 39–77 years of age [average age, 53 years]) and two CD patients (male, aged 35 and 67 years).

### Animal Models

Two DSS experiments were included into the study. Ten C57Bl6N male mice (Charles River, Italy) were exposed to 3% DSS (MP Biomedicals, 38–50 kDa) in drinking water every working day for 3 weeks (5 days a week DSS and 2 days without DSS). Nine control animals were drinking only water. At the beginning of the studies**,** mice were 7–8 weeks old and weighed 19–22 g (median, 21 g). Disease activity in the DSS model was evaluated using a histologic score method published by Nishitani et al. (2009) (Supplementary Table S1B).

Three adoptive transfer experiments have been performed. An adoptive transfer model of chronic colitis was induced in C.B-17 female SCID mice (Charles River, France). At the beginning of the experiments**,** mice were 7–10 weeks old and weighed 15–21 g (median, 18 g). Purified CD4^+^CD25^−^CD62L^+^ T cells were isolated from spleen cells of female BALB/c mice (Charles River) using “CD4+CD25^−^CD62L^+^ T Cell Isolation Kit II, mouse” (Miltenyi Biotec) according to manufacturer instructions. Purified CD4^+^CD25^−^CD62L^+^ cells (>95% pure and >90% viable) were resuspended in sterile phosphate-buffered saline (PBS) (5 × 106 cells/mL) and transferred into SCID mice intraperitoneally (i.p.). Mice with more than 5% of CD4^+^ T cells within total blood leukocytes at day 14 (D14) after transfer were included in the study. Twenty control SCID naive animals and 35 diseased animals were sacrificed at 4, 6, and 8 weeks after transfer. Disease activity in the adoptive transfer model was evaluated using a histologic score method published by Laroux et al. (2004) (Supplementary Table S1C).

### Immunohistochemistry

Immunohistochemistry (IHC) was performed on FFPE tissue using the following primary antibodies: claudin-1 (Abcam, ab56417, 1:250), claudin-2 (Abcam, ab125293, 1:75), claudin-3 (GeneTex, GTX15102, 1:50), claudin-4 (Abcam, ab53156, 1:1,000), and claudin-8 (Abcam, ab183738, 1:1,000) followed by DAKO EnVision Detection System Kit or R&D System Kit (Supplementary Figure S1). Whole slide for each human sample/animal sample was evaluated. Human samples were at least 1 cm long. Animal samples represented the whole colon. Colon was sampled as proximal and distal parts; each part was cut into three to four pieces; whole material was embedded. On one slide, there have been three to four transversal cuts from proximal colon and three to four cuts from the distal colon.

### Quantitative Polymerase Chain Reaction

Total RNA was isolated from the FFPE blocks with the use of RNeasy FFPE Kit, as recommended by manufacturer (Qiagen). First-strand cDNA was obtained by reverse transcription using a commercial SuperScript III First-Strand Synthesis System according to the manufacturer’s protocol (Invitrogen) and random hexamers as primers.

Quantitative real-time polymerase chain reaction (qRT-PCR) for detection of claudin-1, claudin-2, claudin-3, claudin-4, and claudin-8 mRNA expression was performed on an Applied Biosystems 7300 Real-Time PCR system using the TaqMan method and glycerin-aldehyde-3-phosphate (GAPDH) as a housekeeping gene. Only expression of human claudin-3 expression was measured using SyberGreen technology. Primers and probes (Table 1) were designed in Primer Express software version 3.0 and used at validated concentrations. All primers and TaqMan probes were used at a final concentration of 100 nM qRT-PCR was performed in a 30 µL final volume containing 1× Applied Biosystems TaqMan PCR Master Mix or 1× Applied Biosystems SYBR Green PCR Master Mix, 100 nM primers and probes, and 1 µL of cDNA. Amplification was performed using the following cycling conditions: 2 min at 50°C, 10 min at 95°C, and 45 two-step cycles of 15 s at 95°C and 60 s at 60°C. Triplicate qRT-PCR analyses were executed for each sample, and the obtained threshold cycle (CT/Ct) values were averaged. The relative level of tested genes mRNA was then normalized to the GAPDH CT value to give the ΔCT value for each sample.

**TABLE 1:** Sequences of primers and probes used for qPCR.

Human		
Gene		Sequence
GAPDH	F	ACC​CAC​TCC​TCC​ACC​TTT​GAC
	R	CAT​ACC​AGG​AAA​TGA​GCT​TGA​CAA
	P	CTG​GCA​TTG​CCC​TCA​ACG​ACC​A
Claudin-1	F	ACG​AAT​TTG​GTC​AGG​CTC​TCT T
	R	AAG​TAG​GGC​ACC​TCC​CAG​AAG
	P	ACT​GGC​TGG​GCT​GCT​GCT​TCT​CTC T
Claudin-2	F	TCT​GTG​GTG​GGC​ATG​AGA​TG
	R	TGCTACCGCCACTCTGTC TTT
	P	ACA​GTC​TTC​TGC​CAG​GAA​TCC​CGA​GC
Claudin-3	F	AGC​GAG​TCG​TAC​ACC​TTG​CA
	R	CAT​CAT​CAC​GTC​GCA​GAA​CAT
	P	—
Claudin-4	F	GGC​TGC​TTT​GCT​GCA​ACT​G
	R	CAG​AGC​GGG​CAG​CAG​AAT​A
	P	CCA​CCC​CGC​ACA​GAC​AAG​CCT​TAC​T
Claudin-8	F	TGC​AAC​GAA​AAG​AGC​AGT​AGC​T
	R	TTC​CGG​TGT​GAT​AAC​TTT​TTT​GG
	P	CAG​ATA​CTC​GAT​ACC​TTC​CCA​TCG​CAC​AA
Mouse		
Gene		Sequence
GAPDH	F	TGT​GTC​CGT​CGT​GGA​TCT​GA
	R	CCT​GCT​TCA​CCA​CCT​TCT​TGA
	P	CCG​CCT​GGA​GAA​ACC​TGC​CAA​GTA​TG
Claudin-1	F	ATC​TAC​GAG​GGA​CTG​TGG​ATG​TC
	R	AGC​AAG​GAG​TCG​AAG​ACT​TTG​C
	P	CGT​TTC​GCA​AAG​CAC​CGG​GC
Claudin-2	F	GCCCCAGGGCAATCGT
	R	GGA​GAG​CTC​CTA​GTG​GCA​AGA​G
	P	CCA​ACT​ACT​ATG​ATG​GCT​ACC​AGG​CCC​AG
Claudin-3	F	GCGCCTTGCTGTGTTGCT
	R	AGA​GGA​TCT​TGG​TGG​GTG​CAT
	P	CTG​CCC​ACC​GCG​CGA​CAA​G
Claudin-4	F	CGT​GGC​AAG​CAT​GCT​GAT​TA
	R	GTCGCGGATGACGTTGTG
	P	TGC​CCG​TGT​CCT​GGA​CCG​C
Claudin-8	F	GGG​ACG​ATG​AGA​ACG​TGA​AGA
	R	CCA​AGC​CGG​TGA​TGA​AGA​A
	P	CGC​ATC​TTG​CTG​ACA​GCC​GGA​AT

F, forward; P, TaqMan probe; R, reverse.

### Isolation of Lamina Propria Mononuclear Cells From Human Colonic Mucosa, Western Blot Analysis, and Flow Cytometry

Isolation of LPMCs was performed using a lamina propria dissociation kit (Miltenyi Biotec, 130-097-410) following the manufacturer’s instructions. Briefly, the method consisted of epithelial cell removal by incubation of tissue in a solution containing 1 mM DTT and 5 mM EDTA, followed by enzymatic digestion (enzymes included in the kit) and mechanical dissociation using a gentle MACS dissociator (Miltenyi Biotec, 130-093-235). In case of resection specimens, from which higher cell yield was obtained, mononuclear cells were further purified by Lymphoprep density gradient centrifugation. Isolated cells were cryopreserved in medium containing 70% fetal bovine serum (FBS), 20% RPMI 1640, and 10% dimethyl sulfoxide, following the protocol described previously for PBMCs (Kreher et al., 2003).

For Western blot analysis, LPMCs were lysed in PBS with 1% Triton-X (Sigma, T9284), phosphatase inhibitor (Roche, 04906837001), and protease inhibitor (Roche, 04693124001). Protein concentration in lysates was determined using Pierce BCA Protein Assay Kit (23,225; Thermo Fisher Scientific, Waltham, MA, USA) and adjusted to 0.5 mg/mL. Claudin-1, claudin-2, and claudin-4 and GAPDH protein expression was determined by use of Wes System, Wes separation 12–230 kDa capillary cartridges (SM-W004), and standard pack (PS-ST01EZ-8) (all from Protein Simple) according to manufacturer’s instructions. The following primary antibodies were used: GAPDH (Abcam, ab9485, 1:1,000), claudin-1 (Abcam, ab15098, 1:50), claudin-2 (Abcam, ab53032, 1:100) and claudin-4 (Abcam, ab53156, 1:100).

For flow cytometry, LPMCs were thawed and plated in 96-well U-bottom plates for staining. Cells were washed twice with PBS and stained for live/dead discrimination using Invitrogen LIVE/DEAD Fixable Aqua dead cell stain kit (Thermo Fisher Scientific). Blocking of Fc receptors was performed using Human BD Fc Block (Becton–Dickinson, Franklin Lakes, NJ, USA) and was followed by surface staining. Cells were stained with fluorescently labeled monoclonal antibodies directed to claudin-1 BV421 (clone 421203, BD), CD56 BV605 (clone HCD56; BioLegend, San Diego, CA, USA), CD19 BV711 (clone 2H7; BioLegend), CD4 PerCP-Cy5.5 (clone RPA-T4; Thermo Fisher Scientific), claudin-2 PE (polyclonal; Lifespan Biosciences, Seattle, WA, USA), claudin-3 PE (clone REA751; Miltenyi Biotech, Bergisch Gladbach, Germany), claudin-4 PE (clone REA898; Miltenyi Biotech), HLA-DR PE-Cy7 (clone LN3; Thermo Fisher Scientific), CD3 APC (clone SK7; Thermo Fisher Scientific), CD14 AF700 (clone HCD14; BioLegend), and CD45 APC-Cy7 (clone HI30; BioLegend) at 4°C for 30 min. After staining, cells were washed in PBS with 2% FBS and 2 mM EDTA and acquired immediately. Fluorescence-minus-one (FMO) controls were prepared for each claudin. Gates were set using FMO controls with <1% background accepted. Gating strategy for flow cytometry analysis is given in (Supplementary Figure S2). Data were collected using an Attune NxT flow cytometer (Thermo Fisher Scientific) and then analyzed using FlowJo software version 10.7 (FlowJo LLC, Ashland, OR, USA). Graphs were generated using GraphPad Prism software version 9.1 (GraphPad Software, San Diego, CA, USA).

### Statistical Analysis

The Mann–Whitney nonparametric test was used for statistical evaluation of histology score data. Differences in claudin gene expression between healthy and UC or CD patients, as well as between naive animals and animals with induced colitis, were deduced using *t* test. The level of significance was set to *p* < 0.05 in all cases. GraphPad Prism Software (San Diego, CA, USA) was employed.

## Results

### Claudin Expression in Human Colon and IBD

**FIGURE 1:**
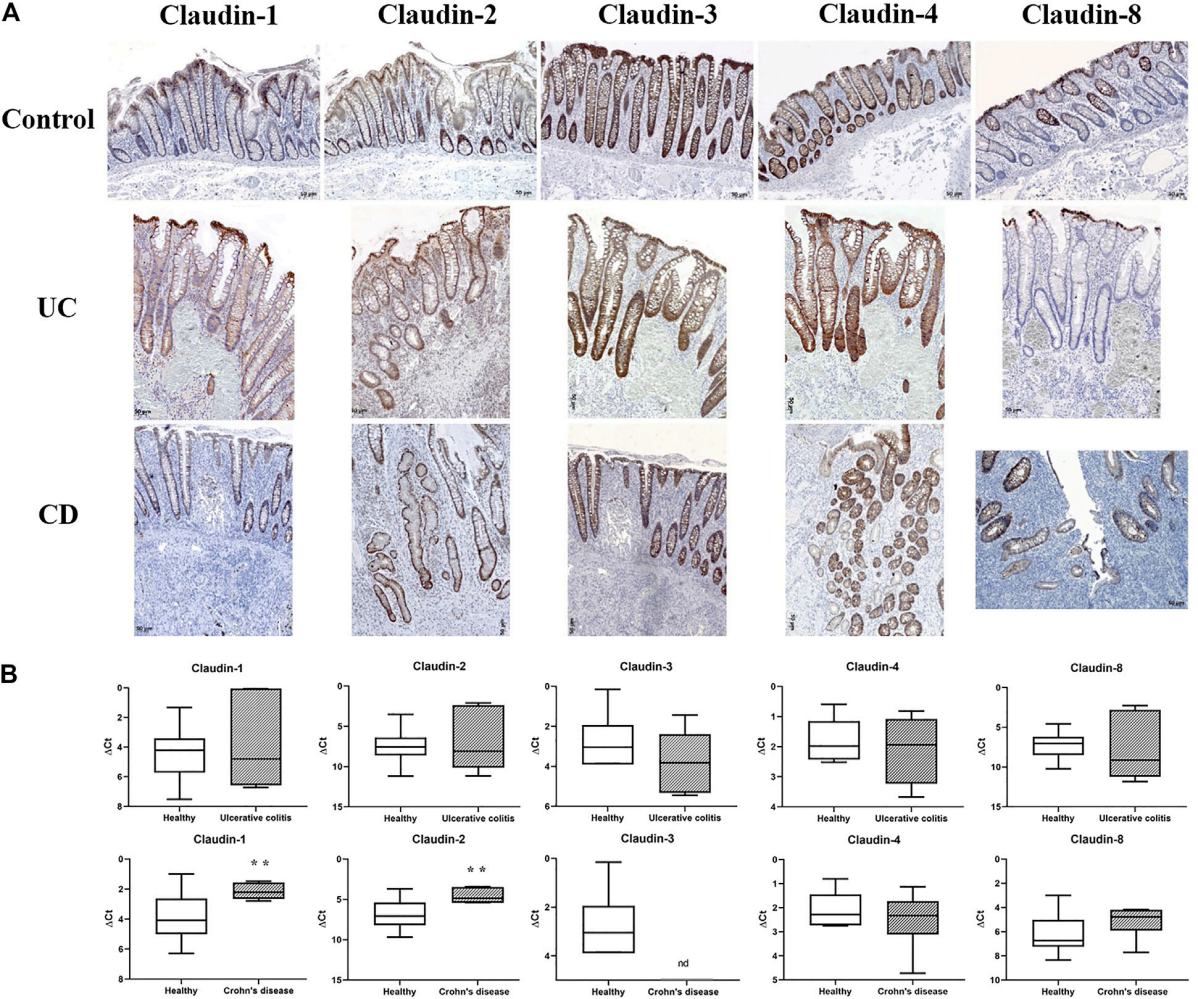
Claudin-1, claudin-2, claudin-3, claudin-4, and claudin-8 expression in control human colon and colon of IBD patients. **(A)** Overview of claudin-1, claudin-2, claudin-3, claudin-4, and claudin-8 expression pattern (IHC); **(B)** claudin mRNA expressed as ΔCT value from claudin CT normalized to the GAPDH CT. Data are presented as box-and-whisker plots, with median and whiskers extending from 5 to 95 percentiles (eight non-IBD, five UC, and eight CD). ND, below detection limit. *t* Test, *p* < 0.05 vs. non-IBD; CD, Crohn disease; UC, ulcerative colitis.

**FIGURE 2:**
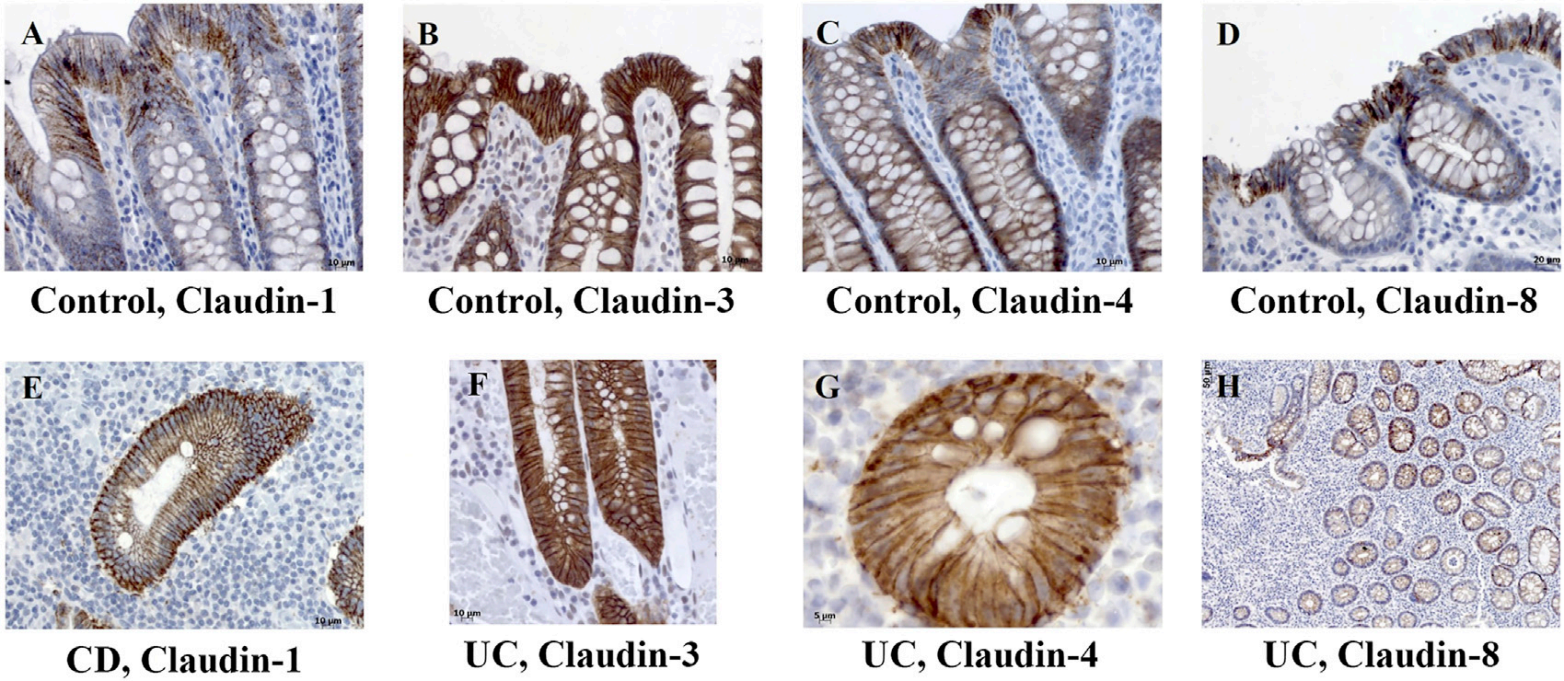
Claudin-1, claudin-3, claudin-4, and claudin-8 expression patterns of absorptive cells in control human colon and colon of IBD patients. **(A)** Claudin**-**1 (IHC), control human colon; **(B)** claudin-3 (IHC), control human colon; **(C)** claudin-4 (IHC), control human colon; **(D)** claudin**-**8 (IHC), control human colon; **(E)** claudin-1 (IHC), CD; **(F)** claudin-3 (IHC), UC; **(G)** claudin-4 (IHC), UC; **(H)** claudin**-**8 (IHC), UC. CD, Crohn disease, UC, ulcerative colitis.

In control human colon, TJ proteins claudin-1, claudin-2, claudin-3, claudin-4, and claudin-8 were variably expressed along the crypt axis and differently distributed along the epithelial cell membrane, reflecting the differences of claudin expression by absorptive and secretory epithelial cells (Figure 1A-Control). Claudin-3 and claudin-4 were expressed along the whole crypt axis, whereas claudin-1 and claudin-8 expression increased toward crypt surface (Figure 1A, Control). Claudin-2 was variably present in colon tissue from different donors and ranged from faint to strong. Absorptive cells expressed claudin-1 (Figure 2A), claudin-3 (Figure 2B), claudin-4 (Figure 2C; Supplementary Figure S3D), and claudin-8 (Figure 2D; Supplementary Figure S3E) at TJs and along basolateral membranes. Claudin-3, claudin-4, claudin-8 (Supplementary Figure S3F), and focally claudin-1 (Supplementary Figures S3B,C) formed TJs of goblet cells. In addition, claudin-3 and claudin-4 (Figures 2B,C), as well focally claudin-1 (Supplementary Figure S3C) and claudin-8, were expressed linearly along the basolateral membrane of goblet cells. Interindividual differences were noted in focal/segmental claudin-1 and claudin-8 expression by goblet cells. Tuft cells were positive for claudin-1 (Supplementary Figure S3A). Apart from epithelial cells, claudins were expressed by cells of mesoderm origin. Claudin-2 was expressed by mononuclear cells in lamina propria, a subset of lymphocytes within lymphoid tissue and endothelial cells. Claudin-3 was expressed by a subset of cells within the germinal center of lymphoid follicles (Supplementary Figures S3G,H) and high endothelial venules’ (HEVs’) endothelial cells (Supplementary Figure S3I).

**FIGURE 3:**
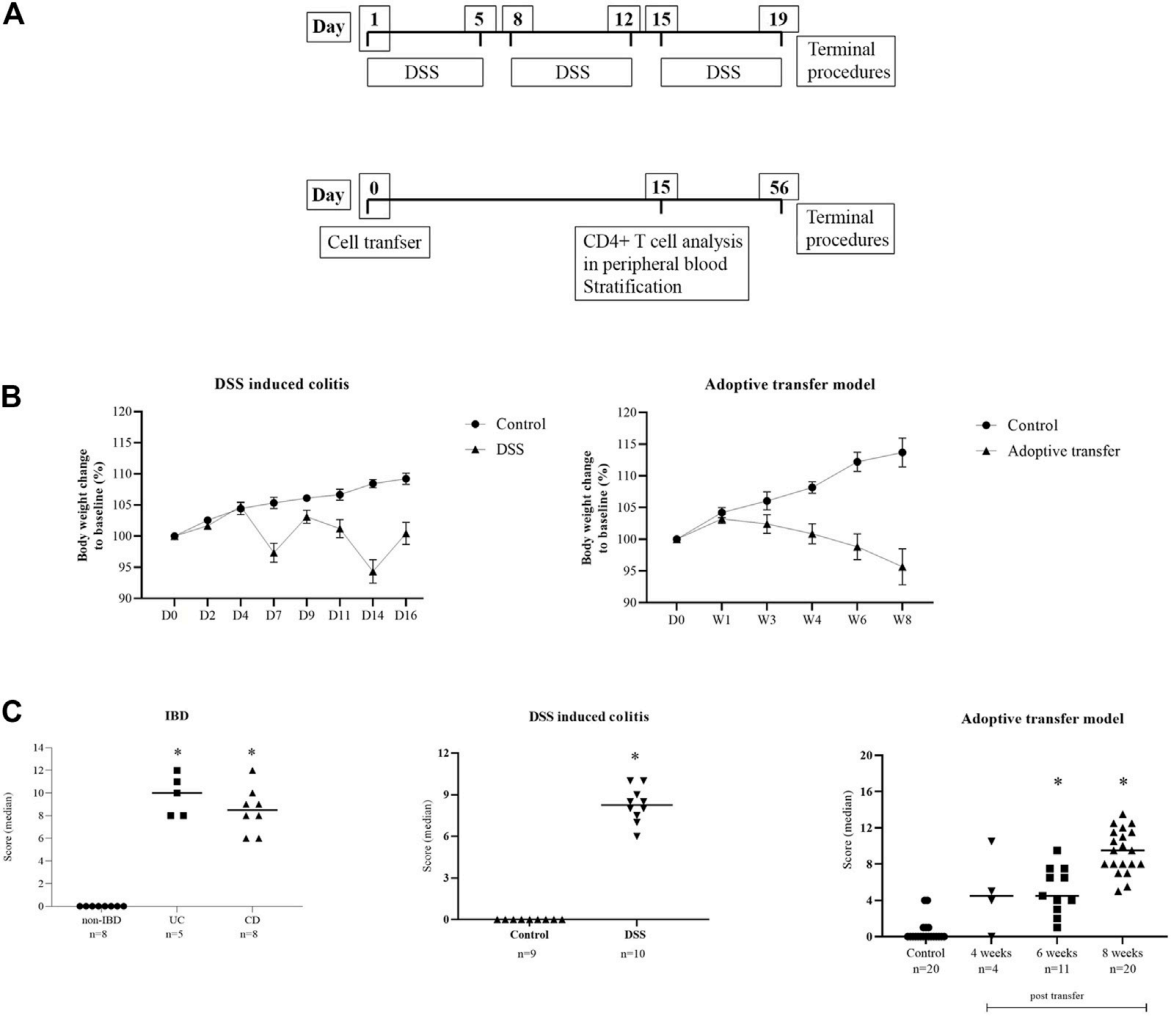
**(A)** Diagrams of the experimental protocols for the induction of DSS colitis and adoptive transfer model in mice. **(B)** Body weight change in animal models. Data are presented as mean ± SEM (D, day; W, week). **(C)** Disease activity score. Score is given as group median; Mann–Whitney nonparametric test; *p* < 0.05 vs. control; IBD (Naini and Cortina, 2012), DSS (Nishitani et al., 2009), adoptive transfer model (Laroux et al., 2004).

**FIGURE 4:**
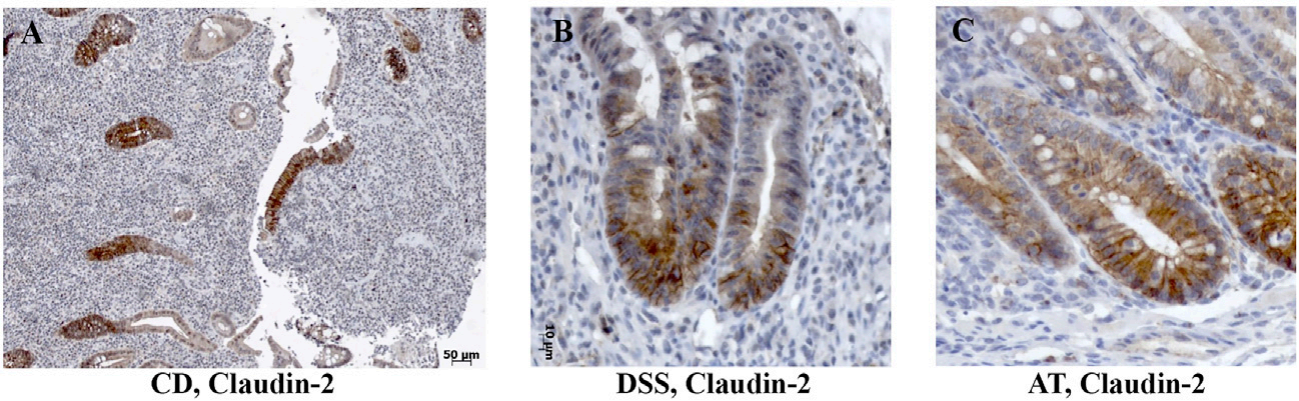
Claudin-2 expression within enlarged proliferative zone in human IBD colon tissue and IBD animal models. **(A)** Claudin-2 (IHC), CD; **(B)** claudin-2 (IHC), DSS-model; **(C)** claudin-2 (IHC); Adoptive transfer model, 4 weeks after transfer. CD, Crohn disease, AT, adoptive transfer model.

**FIGURE 5:**
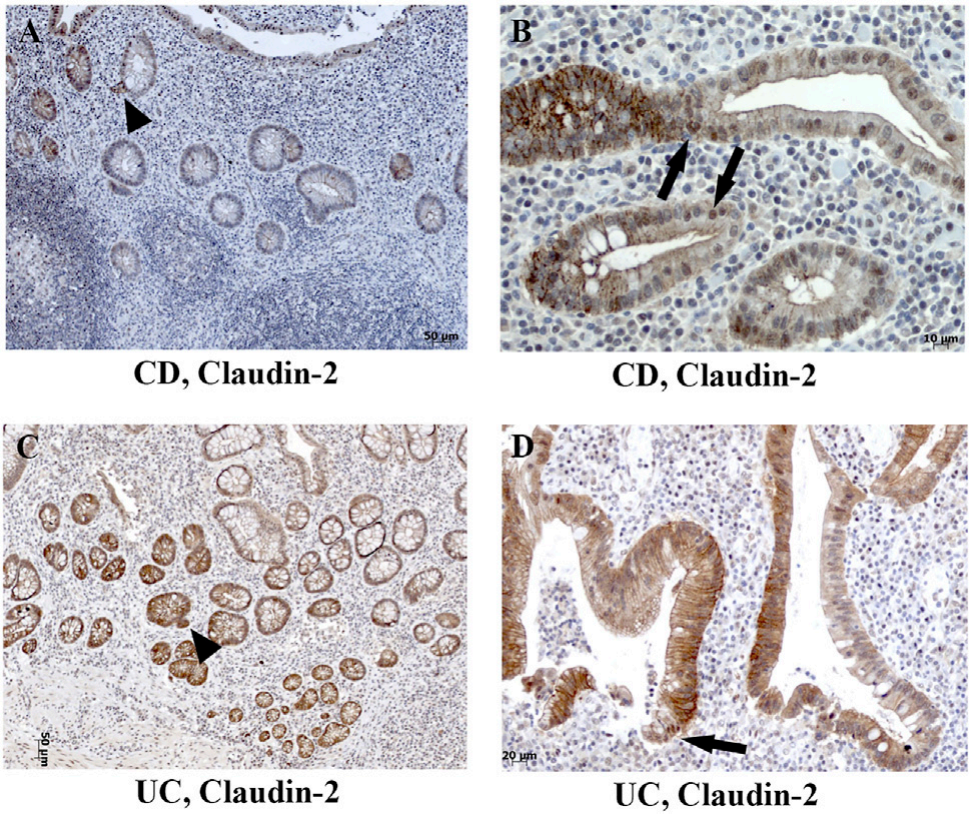
Heterogeneous claudin-2 expression pattern in human IBD colon tissue. **(A)** Claudin-2 (IHC) positive cells at sites of “crypt budding,” CD; **(B)** claudin-2 (IHC) expressed along cell membranes and within nuclei, CD; **(C)** claudin-2 (IHC) positive cells at sites of “crypt budding,” UC; **(D)** claudin-2 (IHC) expressed along membranes and within nuclei, UC. Highlighted features: “crypt budding” (arrowheads) and claudin-2 positive nuclei (arrows). CD, Crohn disease; UC, ulcerative colitis.

**FIGURE 6:**
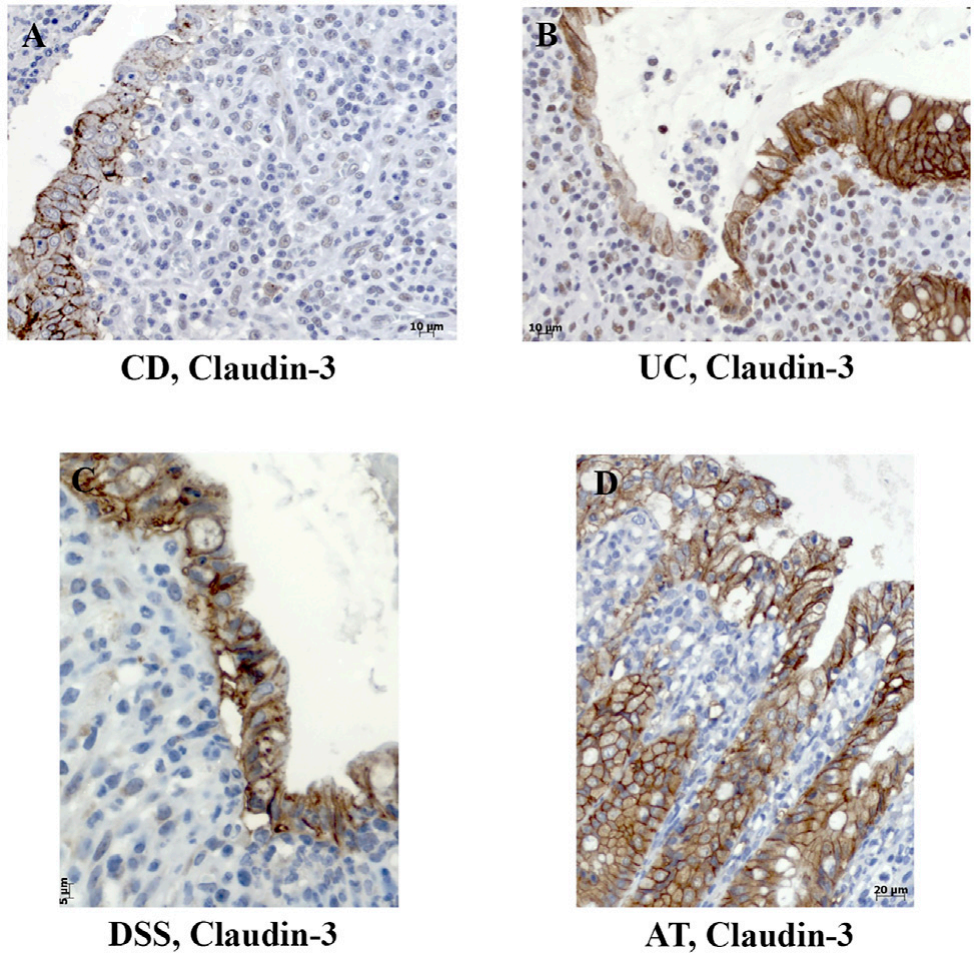
Decreased membranous expression and internalization of claudin-3 in colon tissue of IBD patients and IBD animal models. **(A)** Claudin-3 (IHC), CD; **(B)** claudin-3 (IHC), UC; **(C)** claudin-3 (IHC), DSS; **(D)** claudin-3 (IHC), adoptive transfer model, 8 weeks after transfer. CD, Crohn disease; UC, ulcerative colitis; AT, adoptive transfer model.

In severe IBD cases analyzed in this study (Figure 3C; Supplementary Table S1A), the epithelial proliferation rate and cell differentiation were profoundly disturbed. This was manifested through a plethora of crypt phenotypes, each characterized by a different claudin signature (Figure 1A, UC, CD). Nevertheless, some characteristic patterns were still observed. Lineage claudin expression signature was not altered by the shift of differentiation toward absorptive cells (Figures 2E–H). Claudin-2 was preponderantly, but not exclusively, expressed by cells within an enlarged crypt proliferative zone (Figure 4A) and at sites of “budding” (Figures 5A,C). Claudin-2 expression pattern was heterogeneous and varied among crypts, even between morphologically identical cells within the same crypt (Figures 5B,D). On the other hand, decreased claudin expression by epithelial cells was observed at sites of neutrophil infiltration. Furthermore, intestinal surface epithelium in a vicinity of mucosal erosions and ulcers, in addition to decreased membranous claudin-3 expression, showed internalization of claudin-3 (Figures 6A,B) and claudin-8 into the cell cytoplasm.

**FIGURE 7:**
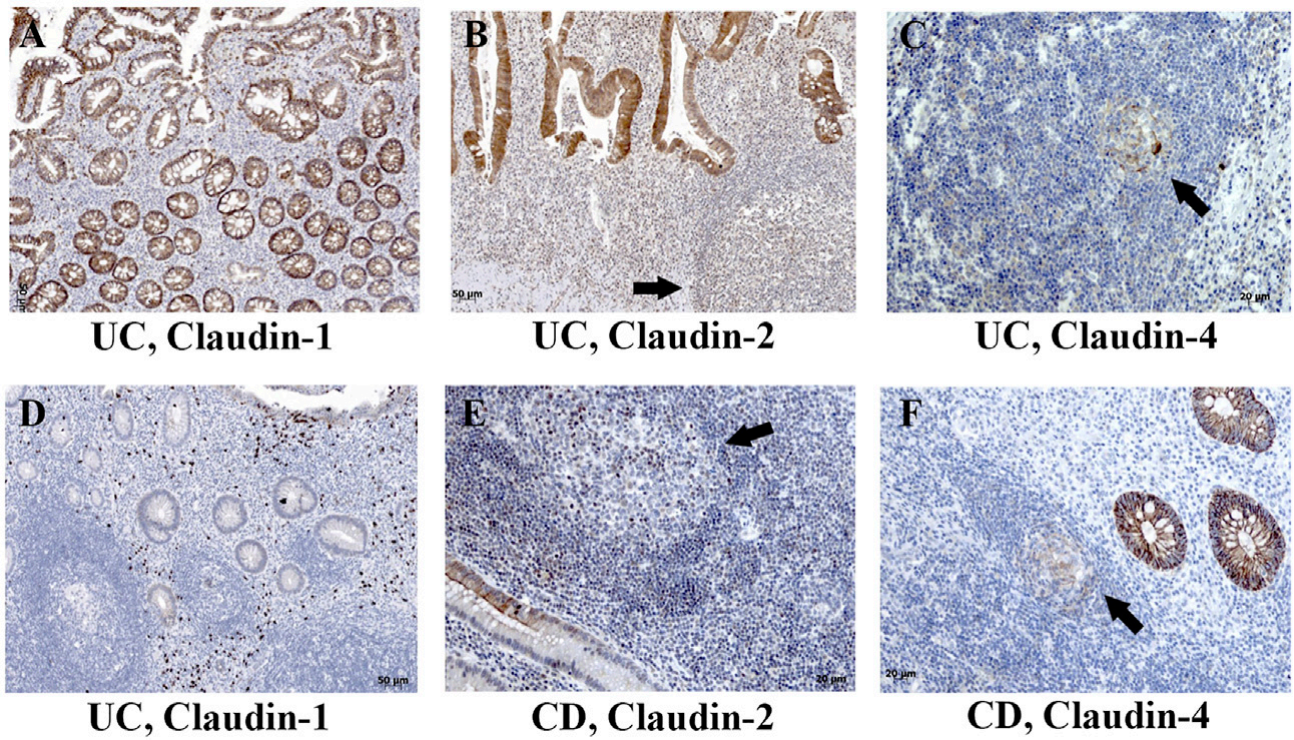
Claudin-1, claudin-2, and claudin-4 expression by mononuclears and within tertian lymph nodes in colon of IBD patients. **(A)** Claudin**-**1 (IHC), UC; **(B)** claudin-2 (IHC), UC; **(C)** claudin-4 (IHC), UC; **(D)** claudin-1 (IHC), UC; **(E)** claudin-2 (IHC), CD; **(F)** claudin-4 (IHC), CD. Highlighted features: claudin positive cells within tertian lymph nodes (arrows). CD, Crohn disease; UC, ulcerative colitis.

**FIGURE 8:**
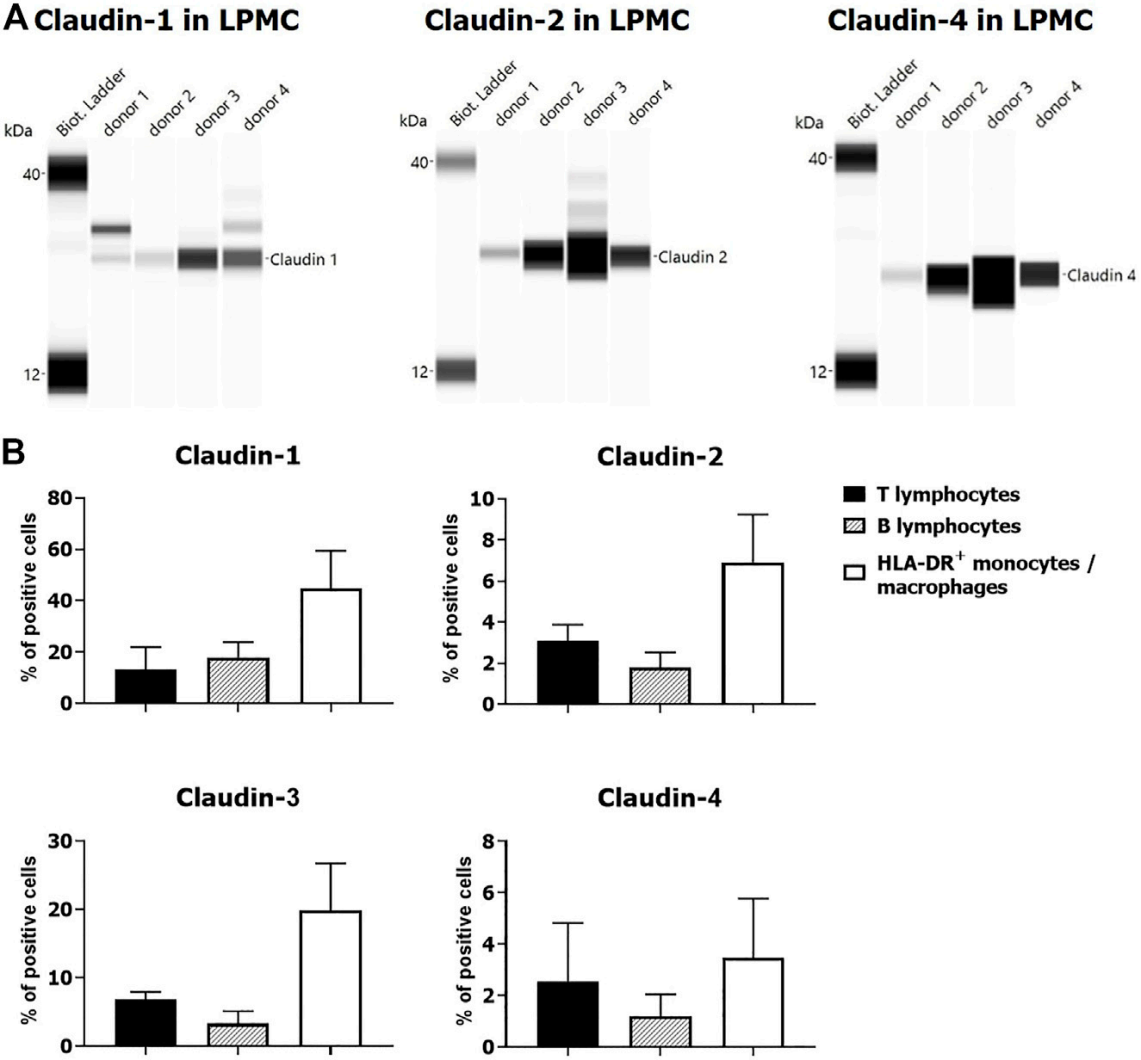
Claudin expression by LPMCs: **(A)** Western blot, four UC donors (colon); **(B)** flow cytometry: percentage of claudin positive cells (mean ± SD) within individual LPMC subpopulations: two UC (colon) and two CD (terminal ileum) donors.

**FIGURE 9:**
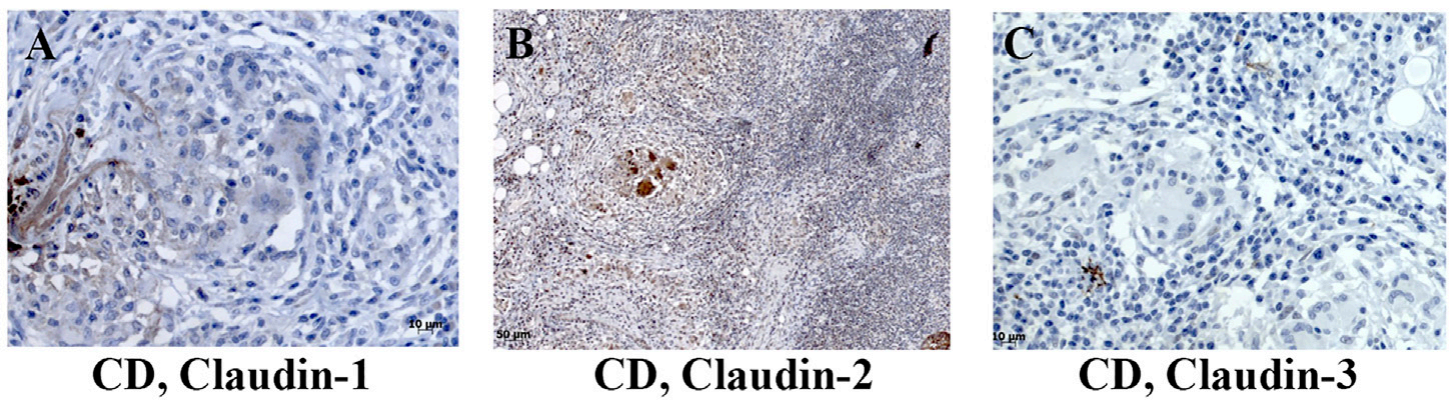
Claudin-1, claudin-2, and claudin-3 expression by granuloma cells in colon of CD patients. **(A)** Claudin-1 (IHC), CD; **(B)** claudin-2 (IHC), CD; **(C)** claudin-3 (IHC), CD. CD, Crohn disease.

Expression of claudin-1 (Figures 7A,D), claudin-2 (Figures 7B,E), and claudin-4 (Figures 7C,F) by inflammatory cells infiltrating into IBD mucosa was noticed in tissue examined by IHC. Protein expression of claudins in isolated LPMCs was confirmed by Western blot analysis and flow cytometry of biopsies obtained from seven IBD donors (Figure 8). Within ectopic tertiary lymphoid tissue, claudin-2 was expressed by lymphocyte subpopulation(s) (Figures 7B,E), and claudin-3– and claudin-4–positive cells were found in germinal centers (Figures 7C,F). In CD, epithelioid macrophages and multinuclear giant cells within granuloma expressed claudin-2 (Figure 9B), while being claudin-1 (Figure 9A) and claudin-3 (Figure 9C) negative.

In surgically removed intestine from patients with CD, claudin-1 and claudin-2 mRNA levels (Figure 1B) were significantly higher than in non-IBD control intestine. Levels of claudin-4 and claudin-8 mRNA remained unchanged (Figure 1B). Claudin-3 mRNA was below detection level in all CD samples. In resected colon samples obtained from patients with UC, claudin-1, claudin-2, claudin-4, and claudin-8 mRNA levels were comparable to non-IBD control intestine (Figure 1B). On the other hand, a tendency toward the lower level of claudin-3 mRNA was observed in UC patients (Figure 1B).

### Claudin Expression in C57Bl/6N Mice and DSS Model

**FIGURE 10:**
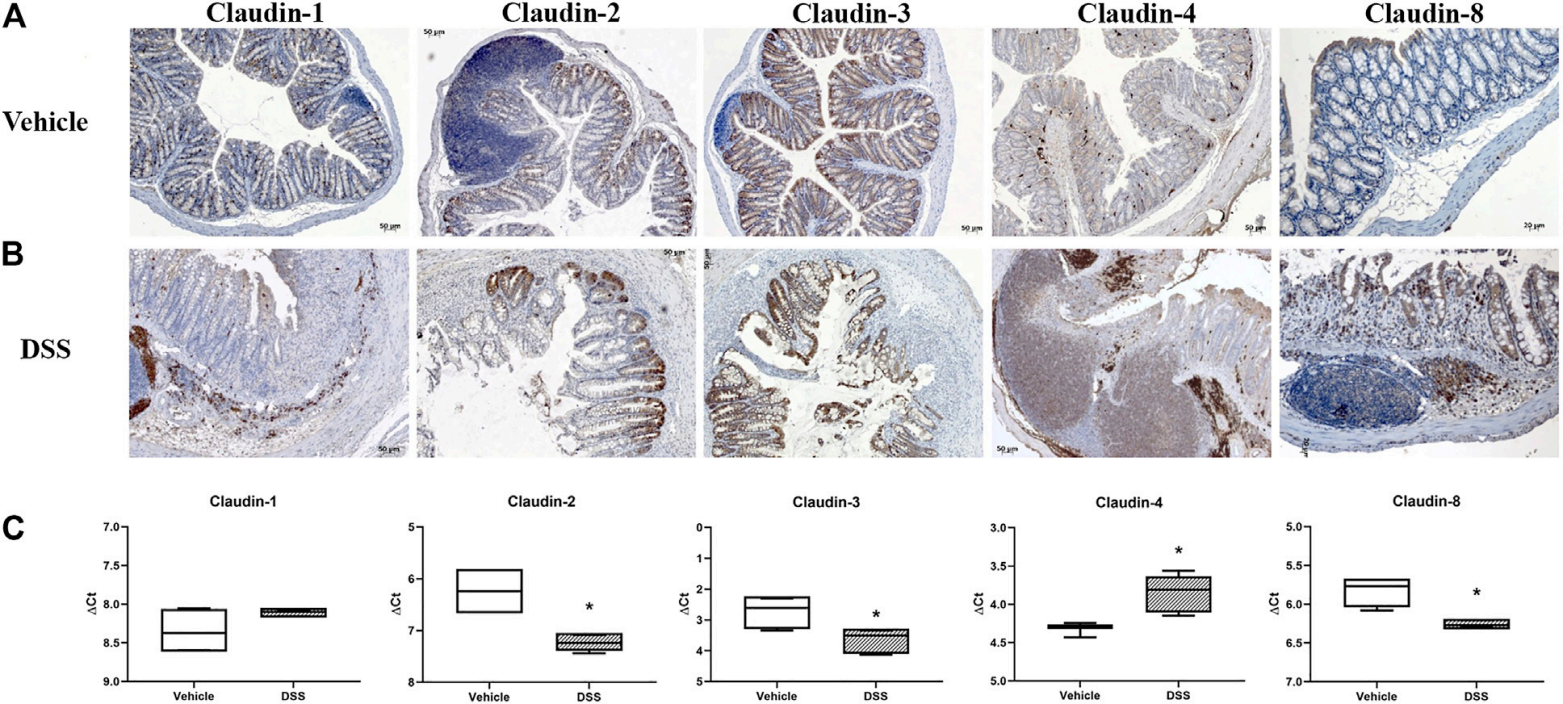
Claudin-1, claudin-2, claudin-3, claudin-4, and claudin-8 expression in DSS-induced colitis, a murine model of IBD. **(A)** Overview of claudin-1, claudin-2, claudin-3, claudin-4, and claudin-8 expression pattern (IHC) in naive C57Bl6N mice; **(B)** overview of claudin-1, claudin-2, claudin-3, claudin-4, and claudin-8 expression pattern (IHC) in DSS-treated mice; **(C)** claudin mRNA expressed as ΔCT value from claudin CT normalized to the GAPDH CT. Data are presented as box-and-whisker plots, with median and whiskers extending from 5 to 95 percentiles (one representative experiment: four naive animals and five animals exposed to DSS). *t* Test, *p* < 0.05 vs. naive mice.

In C57Bl/6N naive colon (Figure 10A), absorptive epithelial cells expressed claudin-3 along lateral membranes, claudin-4 was present at a very low level within the cytoplasm, and claudin-8 was expressed unevenly within the cytoplasm and/or along lateral membranes. Colon absorptive epithelial cells in C57Bl/6N mice did not express claudin-1. Claudin-1 was present within the cytoplasm of crypt secretory cells. All cell types within crypt expressed claudin-3 along the whole length of the basolateral membranes. Claudin-2 was positive in the stem cell niche at the crypt base, situated at TJs and along the basolateral membrane. The claudin-4 expression pattern in crypt was more pronounced in the proliferative region. Claudin-8 was mostly absent from crypt cells. Furthermore, claudin-4 was unevenly expressed by epithelial cells overlying mucosa-associated lymphoid tissue (MALT). The cell subpopulation within MALT expressed claudin-2 and/or claudin-4. The macrophages in lamina propria were strongly claudin-4 positive. Endothelial cells were claudin-2 positive, but claudin-1, claudin-3, claudin-4, and claudin-8 negative.

**FIGURE 11:**
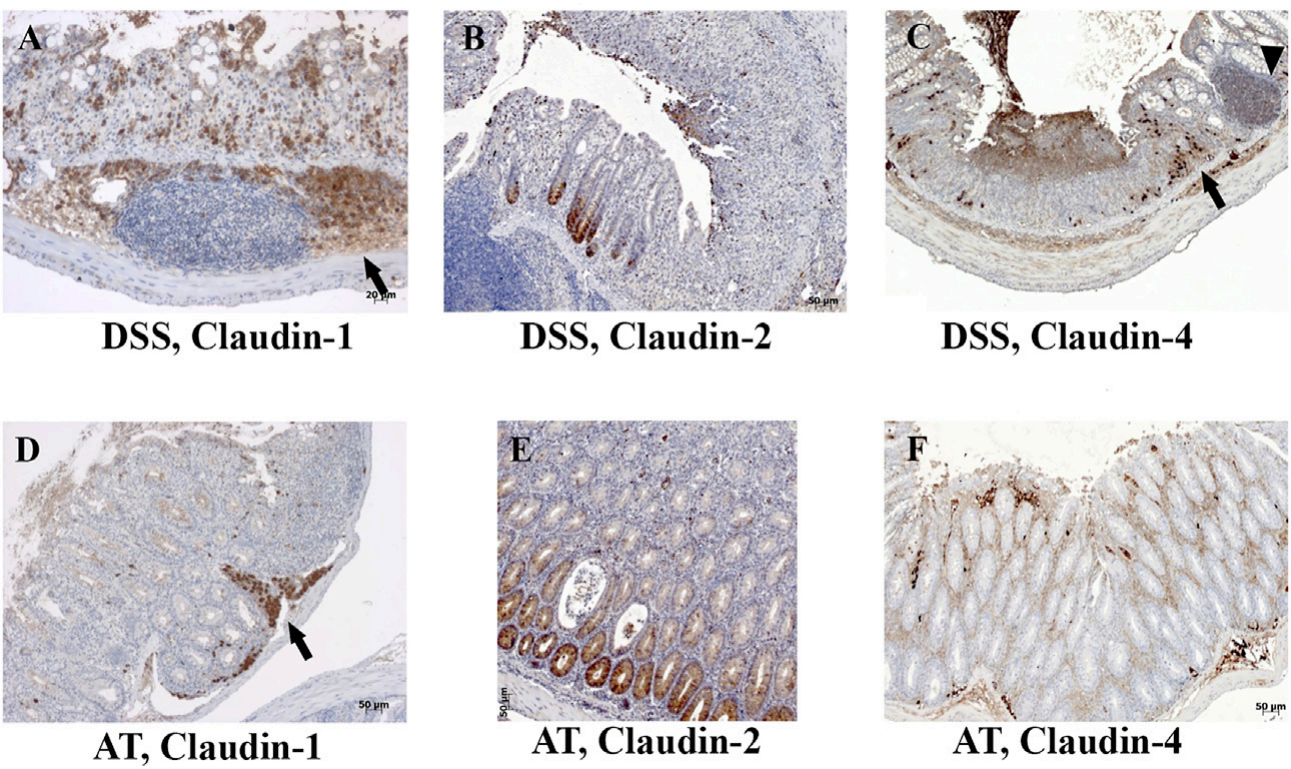
Claudin-1, claudin-2, and claudin-4 expression by mononuclears and within lymph nodes in IBD animal models. **(A)** Claudin-1 (IHC), DSS; **(B)** claudin-2 (IHC), DSS; **(C)** claudin-4 (IHC), DSS; **(D)** claudin-1 (IHC), adoptive transfer, 6 weeks after transfer; **(E)** claudin-2 (IHC), adoptive transfer, 6 weeks after transfer; **(F)** claudin-4 (IHC), adoptive transfer, 8 weeks after transfer. Highlighted features: lymphoid follicle (arrowhead) and accumulation of macrophages (arrows). AT, adoptive transfer model.

DSS-induced murine chronic colitis (Figure 3A) was characterized by weight loss (Figure 3B), colon shortening and chronic active inflammation, mucosa erosions, and formation of ulcers that was reflected through a total histology activity score (Figure 3C, Supplementary Table S1B). Epithelial cells situated at the edge of epithelial defects were claudin-1 and claudin-3 positive along membranes and within the cytoplasm (Figure 6C). In noninflamed mucosa, claudin-3 occasionally formed small intracytoplasmic vacuoles within absorptive epithelial cells. In majority of the epithelial cells, claudin-4 was present at a relatively low level, whereas claudin-8 expression level within epithelial cell compartment increased (Figure 10B). Within the crypt proliferative zone, claudin-2 was overexpressed (Figure 4B; Figure 10B), claudin-3 was localized along lateral membranes, whereas claudin-8 was moderately expressed within the subset of the crypts (Figure 10B). Lymph follicles were strongly claudin-4 positive (Figure 10B; Figure 11C), whereas various infiltrating inflammatory cells in lamina propria were either claudin-1 (Figure 10B; Figure 11A), claudin-2 (Figure 11B), claudin-4 (Figure 11C), or claudin-8 positive (Figure 10B); among those, macrophages expressed claudin-1 (Figure 10B; Figure 11A), claudin-4 (Figure 10B; Figure 11C), and claudin-8 (Figure 10B). qRT-PCR analysis of the mouse colon revealed significantly greater expression of claudin-4 and significantly lower expression of claudin-2, claudin-3, and claudin-8 in DSS-treated mice versus healthy controls when expressed over housekeeping gene (Figure 10C).

### Claudin Expression in SCID Mice and Adoptive Transfer Model of Colitis

**FIGURE 12:**
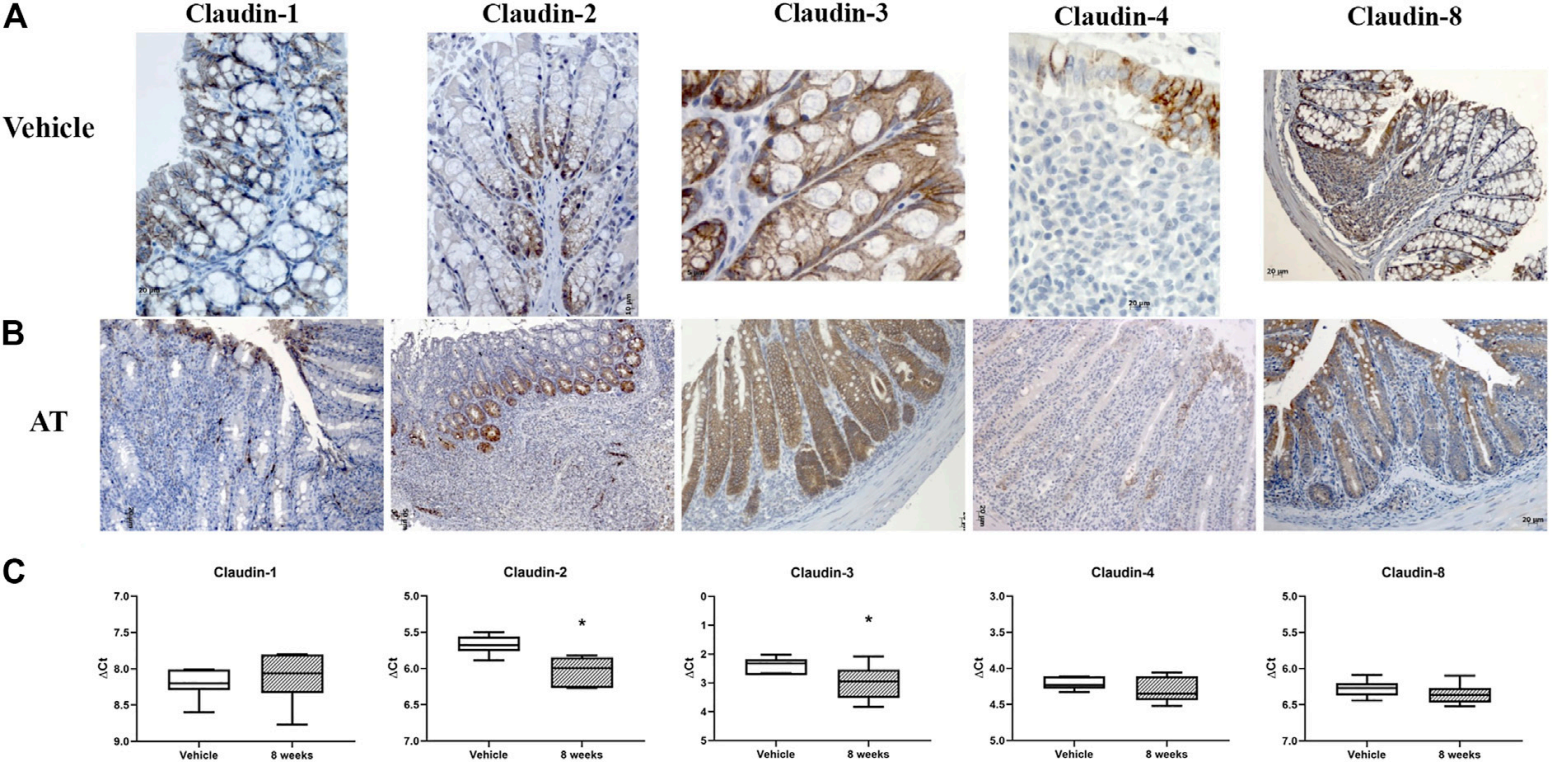
Claudin-1, claudin-2, claudin-3, claudin-4, and claudin-8 expression in adoptive transfer murine model of IBD. **(A)** Overview of claudin-1, claudin-2, claudin-3, claudin-4, and claudin-8 expression pattern (IHC), in naive SCID mice; **(B)** overview of claudin-1, claudin-2, claudin-3, claudin-4, and claudin-8 expression pattern (IHC) in adoptive transfer model, 8 weeks after transfer; **(C)** claudin mRNA expressed as ΔCT value from claudin CT normalized to the GAPDH CT. Data are presented as box-and-whisker plots, with median and whiskers extending from 5 to 95 percentiles (one representative experiment: seven naive animals and nine animals sacrificed 8 weeks after transfer). *t* Test, *p* < 0.05 vs. naive mice; AT, adoptive transfer model.

In SCID murine nondiseased colon, absorptive epithelial cells expressed claudin-1 and claudin-3 along the lateral membranes (Figure 12A). Claudin-8 was unevenly expressed among surface and crypt cells, mainly within the cytoplasm. All crypt cell-types showed membranous claudin-3 expression (Figure 12A). Cells in the proliferative zone, unlike other crypt cells, were strongly claudin-2 positive. Claudin-2 was situated at TJs, along the whole lateral membrane or within the cytoplasm of cells in crypt proliferative zone (Figure 12A). Microfold (M) cells within the epithelium covering the lymphoid tissue expressed claudin-4 along the lateral membranes and occasionally within the cytoplasm, whereas MALT cells were claudin-4 negative (Figure 12A). On the other hand, MALT-associated epithelial cells and lymphoid cells within the MALT were claudin-8 positive (Figure 12A). Claudin-8 was mainly found within the cell cytoplasm. Endothelial cells were claudin-2 positive.

CD4^+^CD25^−^CD62L^+^ T cell transfer into SCID mice induced colitis (Figure 3A) that was manifested by weight loss (Figure 3B), colon elongation, and by mucosal inflammation accompanied by crypt loss due to lymphoid infiltration into the crypt epithelium, diffuse crypt elongation with increased crypt proliferative zone, and reduced goblet cell number (Figure 12B) and was reflected through a total histology activity score (Figure 3C; Supplementary Table S1C). Absorptive cells lining mucosa surface expressed claudin-1 and claudin-3 along the basolateral membranes (Figure 12B). From 4 weeks after transfer onward, cells lining elongated crypts that were lacking goblet cells acquired claudin-1 and claudin-3 expression pattern characteristic for surface absorptive cells (Figure 12B). Most of the surface, crypt body, and crypt base epithelial cells retained cytoplasmic claudin-8 positivity at variable levels (Figure 12B). Claudin-8 translocated toward the membrane in a subset of those cells. In diseased animals, M cells remained claudin-4 positive. Claudin-4–positive cells “outside” MALT-covering epithelium were found. Among epithelial cells covering mucosa surface and in crypts, small clusters of epithelial cells expressed claudin-4 along basolateral membrane (Figure 12B). The crypt proliferative zone was steadily increasing over the posttransfer observation period and was strongly claudin-2 positive (Figure 4C; Figure 11E; Figure 12B). At sites of lymphocytic invasion, epithelial cells lost claudin expression. In adoptive transfer model of IBD subsets of infiltrating mononuclear cells, among other macrophages were claudin-1 (Figure 11D; Figure 12B), claudin-2 (Figure 11E; Figure 12B), claudin-4 (Figure 11F), and/or claudin-8 positive. qRT-PCR analysis of the mouse colon revealed lower expression of claudin-2 and claudin-3 mRNA in mice with adoptive transfer versus healthy controls when expressed over housekeeping gene, whereas claudin-1, claudin-4, and claudin-8 mRNA expression did not significantly change (Figure 12C).

## Discussion

Our study has confirmed that claudin-1, claudin-2, claudin-3, claudin-4, and claudin-8 are constitutive epithelial membrane proteins in nondiseased control human colon with variable distribution pattern along the crypt axis (Lu et al., 2013; Luissint et al., 2016; Garcia-Hernandez et al., 2017). Results obtained in this study highlighted interstrain difference in claudin expression pattern between C57Bl6 and C.B.17/lcr SCID mice (congenic to BALB/c mice; Janvier Labs). Claudin distribution along the crypt axis recorded in either murine strain examined does not completely reflect distribution noted in humans.

Although claudins are primarily regarded as TJ proteins, their distribution outside TJs, along lateral membrane and within the cytoplasm or nucleus of intestinal epithelial cells, in both human and mice was documented in this study. Observed claudin-1 and claudin-3 expression along the whole length of colonocyte basolateral membrane is in line with previous reports in healthy human colon mucosa and IBD, as well as in mice (Prasad et al., 2005; Guttman et al., 2006; Barrmeyer et al., 2015; Hagen, 2017). It has been proposed that claudins situated at basolateral membrane contribute to intestinal homeostasis (Luissint et al., 2016) by forming integrin–claudin adhesion complexes between epithelial cells and extracellular matrix, thus playing a role in cell migration and epithelial–mesenchymal transdifferentiation (Hagen, 2017).

In severe IBD cases analyzed in this study, claudin-2 translocation into the nucleus of epithelial cells was observed. Existence of claudins that shuttle from the membrane to the cytoplasm and even the nucleus has been reported earlier in different malignancies including colorectal adenocarcinoma cells (Dhawan et al., 2005; Ikari et al., 2014; Tokuhara et al., 2017; Torres-Martinez et al., 2017; Hagen, 2017; Chang et al., 2018). To the best of our knowledge, nuclear localization of claudins in nondiseased control intestine or IBD was never reported in the literature. In addition to nuclear claudin expression, increased membranous claudin-2 staining of single cells, as well as increased number of cells expressing claudin-2, was detected regularly in proliferative zone of elongated crypts in resected intestine tissue from IBD patients, as well as in both examined animal models. This phenomenon was described previously by some authors analyzing biopsy tissue from IBD patients (Prasad et al., 2005; Barmeyer et al., 2015; Chang et al., 2018) and adoptive transfer colitis model (Su et al., 2013). It has been shown that apart from contributing to the epithelial barrier function, claudin-2, a Wnt-target gene itself, plays a role in cell proliferation and differentiation. It has been noted that claudin-2–positive cells express Ki67, cyclin D1, and c-Myc (Mankertz et al., 2004; Dhawan et al., 2005; Dhawan et al., 2011; Ikari et al., 2014; Barmeyer et al., 2015; Mah et al., 2016; Ahmad et al., 2017; Torres-Martinez et al., 2017).

In IBD patients and in both animal models studied, the presence of crypts lined by a single epithelial cell type, characterized by strain-specific claudin expression pattern of either absorptive or goblet cells, was observed. Differentiation of intestinal cells into absorptive and secretory cell lineage is regulated, among others, by Notch signaling pathway (VanDussen et al., 2012; Bankaitis et al., 2018). Certain claudins can influence Notch pathway, and in return, claudin expression level lies under Notch control. It has been reported that claudin-1 regulates Notch signaling, thus influencing shift toward absorptive cell lineage differentiation (Pope et al., 2014). Loss of Notch-1 signaling induced claudin-2 expression (Dahan et al., 2011) and differentiation shift toward secretory lineage (VanDussen et al., 2012).

In inflammatory milieu, claudin expression level/pattern by epithelial cells was altered. Although CD and UC do not entirely share pathophysiology, with CD being characterized by granulomatous inflammation, whereas UC is T_H_2-driven (Ramos and Papadakis, 2019), certain similarities are present. One of the common IBD hallmarks is neutrophilic inflammation and mucosal defects due to necrosis, ranging from erosions to ulcer formation. In examined IBD cases, neutrophilic infiltration of mucosa was accompanied by a lower expression of claudin by enterocyte, a phenomenon that was described in IBD previously by Kucharzik (2001). In IBD patient and animal model colon tissue, not only a decrease of claudin expression level has been documented, but also a disruption of continuous claudin-3 expression along the lateral membranes and its internalization into the cytoplasm of cells in the vicinity of mucosal defects in the IBD and DSS model. Translocation of certain claudins from the enterocyte membrane into the cytoplasm was observed in a murine model of necrotizing colitis induced by commensal bacteria (Bergmann et al., 2013). It has also been shown that claudin-1 and claudin-3 relocate from the membrane into the cell cytoplasm following infection with certain pathogens (Guttman et al., 2006). In particular, *Clostridium perfringens* enterotoxin is able to bind claudin-3 and induce its internalization (Luissint et al., 2016).

In the adoptive transfer model, a lack of claudin epithelial staining at sites of intraepithelial lymphocyte infiltration was noted. Loss of claudin expression could be linked to the inflammatory environment. It is known from the *in vitro* studies that cytokines can alter epithelial claudin expression and hence influence permeability of intestinal epithelial barrier (Albert-Bayo et al., 2019). Tumor necrosis factor (TNF-α), interleukin 1β (IL-1β), and interferon γ, all playing a role in IBD (Guan and Zhang, 2017; Ramos and Papadakis, 2019) and IBD animal models (Lyons et al., 2018; Yan-Hong et al., 2018), downregulate claudin-3 and claudin-4 expression by epithelial cells, whereas IL-23 and IL-9 downregulate claudin-8 (Prasad et al., 2005; Zhu et al., 2019). On the other hand, TNF-α, IL-6, IL-13, and IL-17 upregulate claudin-2 expression (Kinugasa et al., 2000; Prasad et al., 2005; Weber et al., 2010), thus increasing barrier permeability. As discussed previously, upregulation of claudin-2 expression by cytokines could influence not only the function of the epithelial barrier, but also cell proliferation.

In nondiseased SCID mice, claudin-4 was exclusively expressed by the follicle-associated epithelium covering MALT, known as M cells. In C57Bl/6 mice, M cells were claudin-4 positive, as previously reported (Tamagawa et al., 2003), but this was not such a striking characteristic as in SCID mice, since in C57Bl/6 mice, other epithelial cells were also expressing claudin-4. M cells are specialized for antigen uptake from the intestine lumen into lymphoid tissue (Nakamura et al., 2018) and have a role in the development of gut immune tolerance and microbial-pathogen defense (Jung et al., 2010). Induction of claudin-4–positive epithelial cells apart from MALT-covering epithelium, but not necessarily overlying lymphoid infiltrate, was evident after T-cell transfer in the adoptive transfer model. Such small clusters of claudin-4 epithelial cells were found also in the DSS model. The true lineage of these cells, absorptive versus M-cell, cannot be deduced from the results of our study.

In addition to claudin expression by epithelial cells, we could confirm claudin expression by cells originating from mesoderm, both in humans and mice. The claudin expression pattern differed among examined species in healthy and diseased intestine. In our report, we concentrated on claudin-expression by endothelial and myeloid cells. Specific claudin signatures were documented for different species and vessel-type endothelial cells. In humans, C57Bl/6 and SCID mice endothelial cells of colon vessels expressed claudin-2. On the other hand, HEVs in humans were claudin-3 positive, while negative in both murine strains. These data are in concordance with an earlier publication on claudin expression on endothelial/HEV cells in C57Bl/6 mice (Pfeiffer et al., 2008) and support literature reports on the lack of overlap among claudin signatures of various endothelial cell types (Komarova et al., 2017). Among claudins studied in our work, claudin-2 was identified as the most widely expressed among cells of myeloid origin in human colon. Claudin-1–, claudin-2–, and claudin-4–positive mononuclear cells in human IBD colon mucosa have been documented by IHC. Protein expression of claudins was confirmed by Western blot analysis and flow cytometry of LPMCs isolated from colon mucosal samples obtained from IBD patients. Furthermore, we also report that epithelioid macrophages and multinucleated giant cells, found within granuloma in CD, express claudin-2. Monocyte-derived macrophages and tissue resident macrophages can transform into epithelioid macrophages under the influence of various cytokines and consequently fuse to form multinucleated giant cells (Timmermans et al., 2016; Brooks et al., 2019). Our findings are, to a certain extent, in line with earlier reported claudin-2 expression by CD163-positive macrophages in noncancerous human colon mucosa (Mezheyeuski et al., 2018). Apart from macrophages, dendritic cells were reported to express claudin-1 and claudin-2 (Rescigno et al., 2001; Van den Bossche et al., 2012). In contrast to humans, lymphocytes in colon lamina propria of naive C57Bl/6N and SCID mice did not express any of the studied claudins. Claudin-2– and claudin-4–positive cells within MALT tissue were found in C57Bl/6N, but not SCID mice. In both IBD models examined in our study, infiltrating myeloid cells, among others macrophages, showed claudin-1, claudin-2, claudin- 4, and claudin-8 positivity. Claudin-1, claudin-2, and claudin-8 expression by alternatively activated macrophages was observed *in vitro* and in murine *in vivo* models (Van den Bossche et al., 2012). In this study, various claudin expression patterns by diverse myeloid cell types in IBD and IBD-models have been documented, but it is beyond the scope of our work to discuss their role upon activation under different inflammatory stimuli.

Next to the examination of claudin expression by IHC, we have evaluated claudin mRNA expression in neighboring tissue slices of the same paraffin blocks. In this study, increased claudin-1 and claudin-2 mRNA levels in colon resection specimens from patients with severe CD were observed. Oshima et al. (2008) reported increased claudin-2 mRNA level in rectal biopsies from UC patients. Differently to observed increase in claudin-2 mRNA expression in human IBD, a decrease of claudin-2 mRNA was observed in both animal models. An increase of claudin-1 mRNA expression observed in CD patient colon tissue was not detected in either of animal models, whereas an increase of claudin-4 mRNA and decrease of claudin-8 mRNA were observed only in the DSS model. A decrease of claudin-3 mRNA detected in IBD patient colon tissue and tissue from both animal models was in line with observed histological changes reflecting relative decrease of claudin-3–positive epithelial cell population versus claudin-3–negative myeloid cell population. Based on the results gained in this study, it can be stated that changes in claudin mRNA level are not a mere consequence of alteration in claudin expression by epithelial cells (Mennigen et al., 2009), as an influx of claudin-1–, claudin-2–, and claudin-4–positive inflammatory cells into mucosa was documented by IHC, Western blot, and flow cytometry. Changes in claudin mRNA isolated from the whole tissue samples reflect overall dynamic of claudin protein synthesis within epithelial and lamina propria compartment: loss of epithelial cells, proliferation of epithelial cells, influx of claudin positive/negative inflammatory cells, and finally relative proportion of certain cell types in analyzed tissue samples. As underlined in this study, mRNA level should be interpreted based on tissue morphology.

Claudins are emerging drug targets; thus, evaluation of their expression in human disease and animal models is crucial for the scientists working in the field of drug discovery. Results of the presented study emphasize the variety of claudin expression patterns among cells of epithelial and myeloid lineage, thus urging for overwhelming and throughout investigation of potential drug effects on claudin expression/function in a wide spectrum of cells in inflamed intestine that is not based solely on quantitative measure of mRNA/protein level within tissue or isolated cell population. Even the alteration of claudin expression in the single cells within highly organized structures such as lymph follicles could have profound downstream consequences for the disease development. Therefore, 1) claudins should not be regarded solely as TJ proteins involved exclusively in the intestinal barrier function, and expression of claudins by inflammatory cells in inflamed mucosa should be considered; 2) differences and similarities in claudin expression pattern by intestinal epithelial cells, in noninflamed and inflamed mucosa, between humans and mice, as well as between murine strains and animal models, should be respected; 3) results of quantitative measurements of claudin level in the tissue should be interpreted with caution and in the context of tissue alterations.

In summary, claudin expression pattern in uninflamed and inflamed colon varies between species, but at the same time, some common characteristics are noted. Claudins are not expressed solely by epithelial cells and are not exclusively TJ proteins. Claudin expression by LPMCs has been confirmed by IHC, flow cytometry, and Western blot. Noted changes at claudin mRNA tissue level in IBD and in either model reflected complex alterations in claudin expression by epithelial cells and relative relation between claudin-positive epithelial cells and claudin bearing inflammatory cells.

## Data Availability

The original contributions presented in the study are included in the article/Supplementary Material, further inquiries can be directed to the corresponding author.

## Supplementary Material

Supplementary Figure S1 Negative control for immunohistochemistry: tissue stained without primary antibody. **(A)** R&D System Kit CTS002, control mouse tissue; **(B)** R&D System Kit CTS005, control mouse tissue; **(C)** R&D System Kit CTS008, control mouse tissue; **(D)** DAKO EnVision System Detection Kit, Human control tissue

Supplementary Figure S2 Gating strategy for flow cytometry analysis. Time vs. forward scatter-height (FSC-H) was used to check the quality of the sample acquire followed by exclusion of doublets on FSC-H vs FSC- Width (FSC-W) and FSC-H vs FSC-Area (FSC-A). Side scatter-area (SSC-A) vs red-FL3-A APC-Cy7 CD45 was done to gate CD45^+^ leukocytes. From live CD45^+^ leukocytes gate B and T lymphocytes were gated followed by a gate from double-negative population (CD20^−^CD3^−^) was used for gating HLA-DR^+^CD14^+^ monocytes/macrophages cells. Each claudin (claudin-1, claudin-2, claudin-3, and claudin-4) was gated on histogram plots for each cell type population. Fluorescence-minus-one (FMO) controls were used for setting the gates.

Supplementary Figure S3 Claudin-1, claudin-4, and claudin-8 expression in absorptive/ secretory epithelial cells and claudin-3 expression within lymphoid follicle in control human colon tissue. **(A)** Claudin-1 (IHC), control, tuft cells; **(B)** claudin-1 (IHC), control, goblet cells; **(C)** claudin-1 (IHC), control, goblet cells; **(D)** claudin-4 (IHC), control, absorptive cells; **(E)** claudin-8 (IHC), control, absorptive cells; **(F)** claudin-8 (IHC), control, goblet cells; **(G)** claudin-3 (IHC), control, epithelium, and lymphoid follicle; **(H)** claudin-3 (IHC), control, germinal center; **(I)** claudin-3 (IHC), control, HEV.

Supplementary Table S1 Criteria for histological evaluation of disease activity in IBD and animal models of IBD. **(A)** Histologic score for disease activity in IBD patients modified from the method published by Naini and Cortina (2012) **(B)** Histologic score for disease activity in DSS animal model published by Nishitani et al. (2009) **(C)** Histologic score method for disease activity in adoptive transfer animal model published by Laroux et al. (2004).
