# Supplementary figures and images for: Claudins: Beyond Tight Junctions in Human IBD and Murine Models
Source: Front Pharmacol. 2021 Nov 17;12:682614. doi: 10.3389/fphar.2021.682614 (PMC8635807; doi:10.3389/fphar.2021.682614)

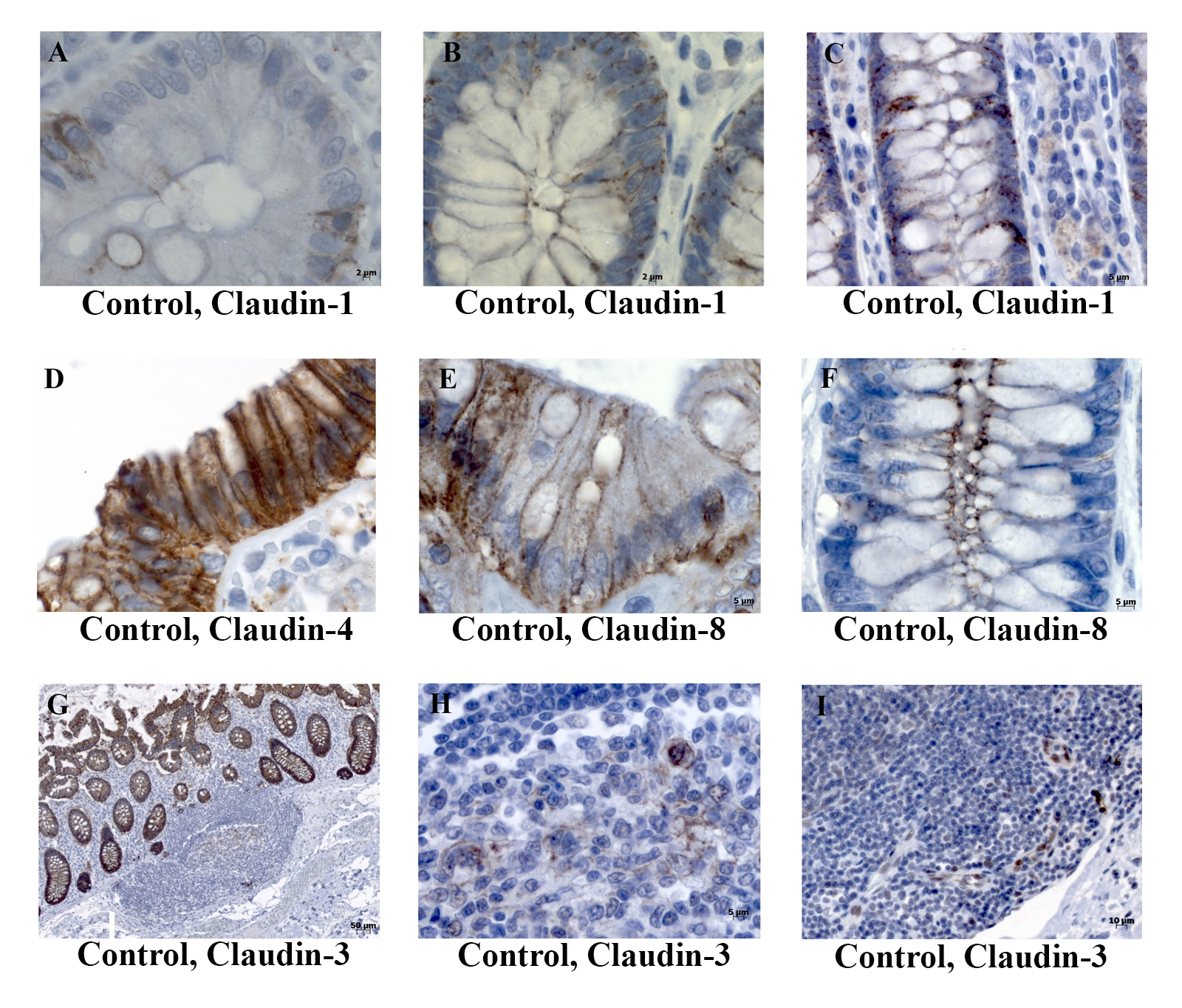

Supplement: Supplementary file 1 [file Image3.JPEG]

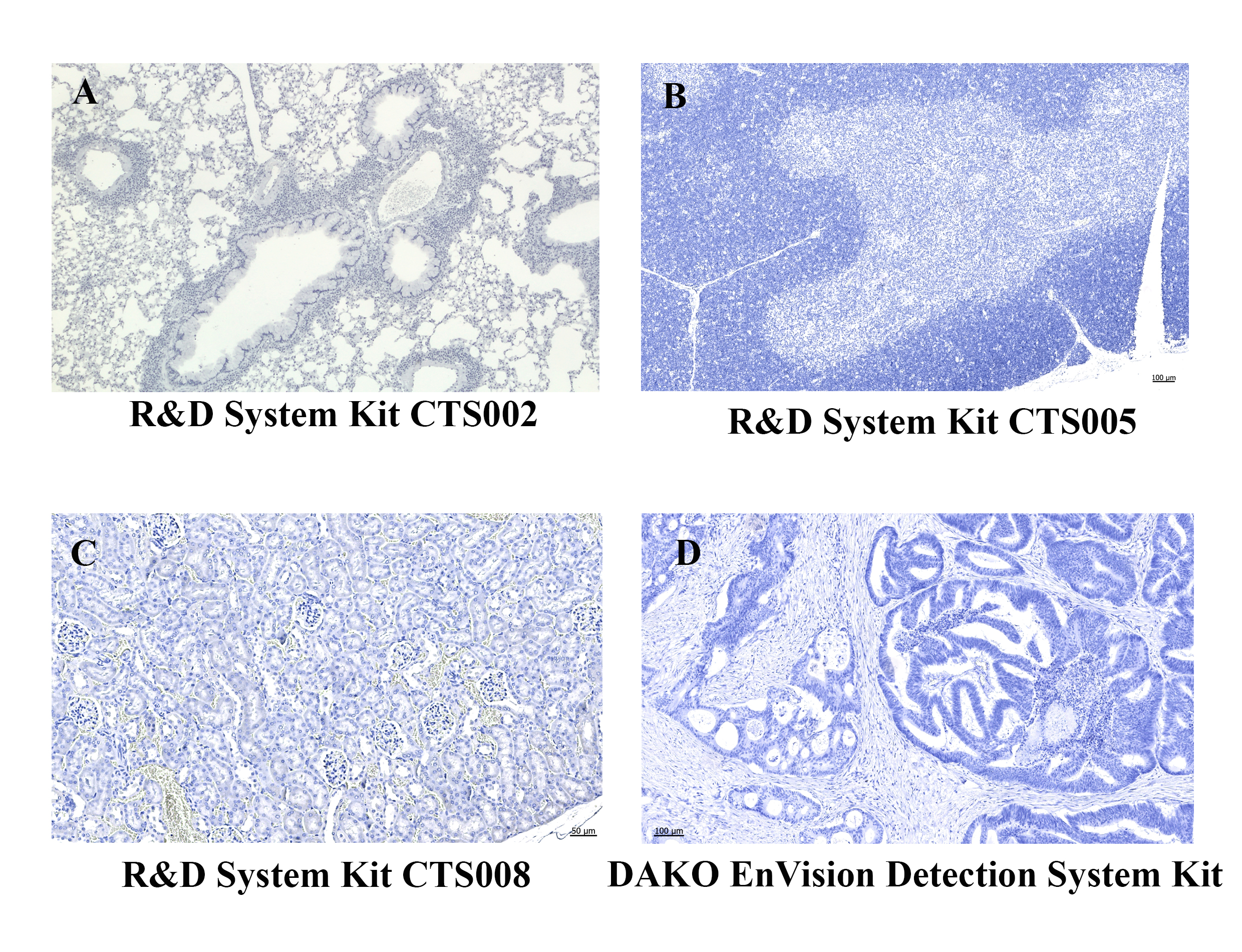

Supplement: Supplementary file 3 [file Image1.JPEG]

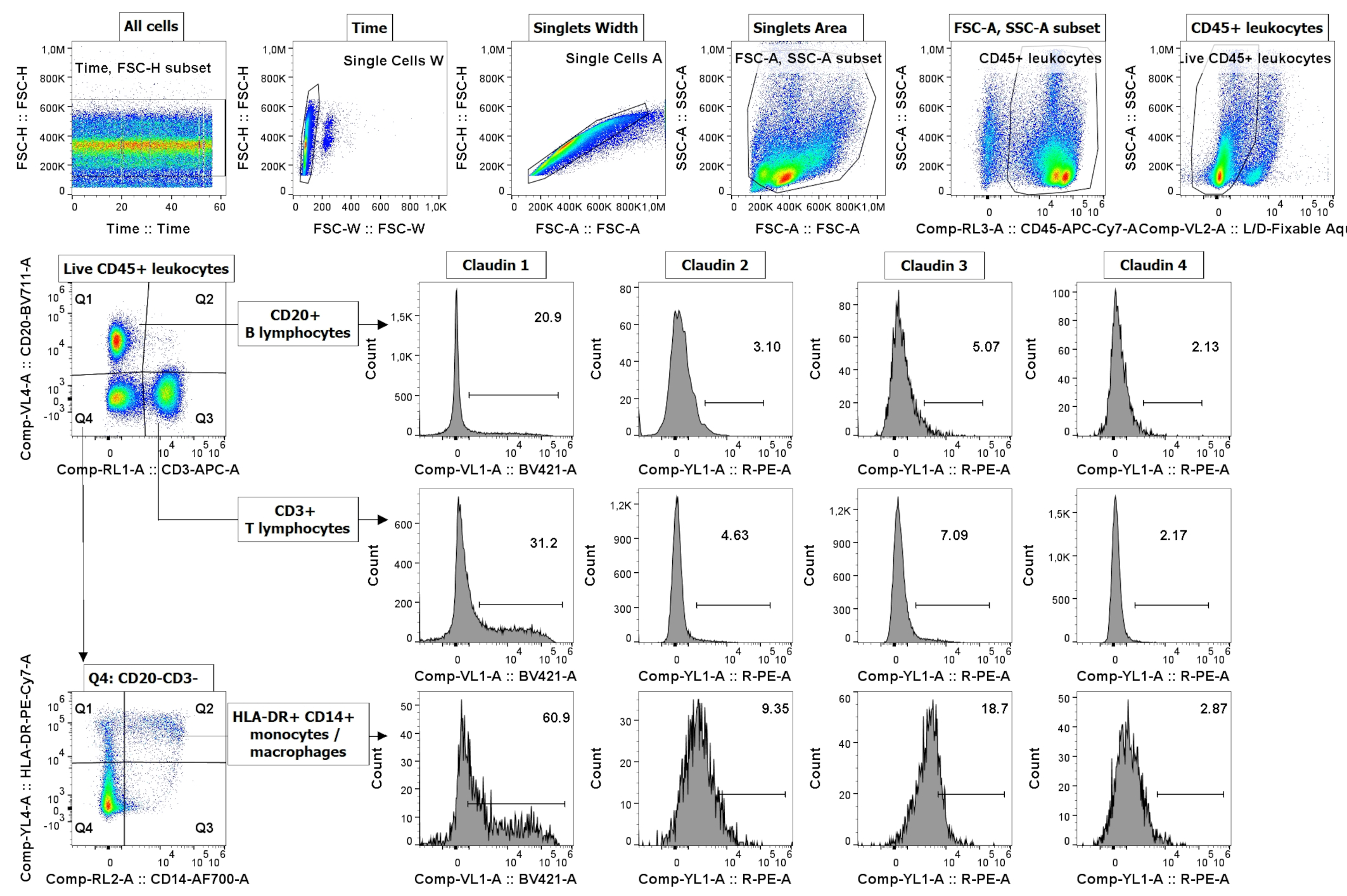

Supplement: Supplementary file 4 [file Image2.JPEG]
