# Supplementary material for: Claudins: Beyond Tight Junctions in Human IBD and Murine Models
Source: Front Pharmacol. 2021 Nov 17;12:682614. doi: 10.3389/fphar.2021.682614 (PMC8635807; doi:10.3389/fphar.2021.682614)
Supplement: Supplementary file 2 [file Table1.pdf]

Supplementary Table 1A Evaluation of disease activity in IBD patients

COLON	Crypt architecture distortion	Basal lymphoplasma cytosis	Cryptitis/ crypt abscesses	Ulcers	Paneth cell or pyloric metaplasia	Granulomas	Increased eosinophils in lamina propria	Lymphoid nodules at base	Muscularis mucosae hyperplasia
0	Absent	Absent	Absent	Absent	Absent	Absent	Absent	Absent	Absent
1	Mild	Mild	Mild	Present	Present	Present	Mild	Present	Present
2	Conspicious	Conspicious	Conspicious	x	x	x	Conspicious	x	x

Modified from published (Naini and Cortina, 2012) histologic score method for disease activity in IBD patients

Supplementary Table 1B Evaluation of disease activity in DSS animal model of IBD

COLON	Severity of inflammation	Layers involved	Epithelial damage	Extent
0	No inflammation	No inflammation	Intact epithelium	No lesions
1	Mild	Mucosa	Disruption of the architecture	Punctuate
2	Moderate	Mucosa and submucosa	Erosion	Multifocal
3	Severe	Transmural	Ulceration	Diffuse

Histologic score method for disease activity in DSS animal model (Nishitani et al. (2009))

Supplementary Table 1C Histologic score for disease activity in adoptive transfer animal model of IBD

COLON	Degree of inflammatory infiltrate	Loss of goblet cells	Reactive epithelial hyperplasia	Number of intraepithelial lymphocytes	Abnormal crypt architecture	Number of crypt abscesses	Mucosal erosion	Submucosal spread
0	No inflammation	No alterations	No alterations	None	No abnormalities	None	None	None
1	Mild	Goblet cells still present	Increased number of mitoses	Mild	Crypt distortion, elongation, branching	Mild	Small/ rare superficial erosions	Submucosa
2	Moderate	Goblet cells absent/ almost absent	“Curly” epithelium	Moderate	Atrophy	Moderate	Large/deep /diffuse erosions	Transmural spread
3	Severe	x	Polyps	Severe	Crypt loss	x	x	x

Histologic score method for disease activity in adoptive transfer animal model (Laroux et al. (2004))
